# The DNA methylome in panic disorder: a case-control and longitudinal psychotherapy-epigenetic study

**DOI:** 10.1038/s41398-019-0648-6

**Published:** 2019-11-21

**Authors:** Christiane Ziegler, Franziska Grundner-Culemann, Miriam A. Schiele, Pascal Schlosser, Leonie Kollert, Marina Mahr, Agnieszka Gajewska, Klaus-Peter Lesch, Jürgen Deckert, Anna Köttgen, Katharina Domschke

**Affiliations:** 1 Department of Psychiatry and Psychotherapy, Medical Center – University of Freiburg, Faculty of Medicine, University of Freiburg, Freiburg, Germany; 2 Institute of Genetic Epidemiology, Faculty of Medicine and Medical Center – University of Freiburg, Freiburg, Germany; 3 Department of Psychiatry, Psychosomatics and Psychotherapy, University of Würzburg, Würzburg, Germany; 4 Division of Molecular Psychiatry, Center of Mental Health, University of Würzburg, Würzburg, Germany; 5 Laboratory of Psychiatric Neurobiology, Institute of Molecular Medicine, I.M. Sechenov First Moscow State Medical University, Moscow, Russia; 6 Department of Neuroscience, School for Mental Health and Neuroscience (MHeNS), Maastricht University, Maastricht, The Netherlands; 7 Center for Basics in NeuroModulation, Faculty of Medicine, University of Freiburg, Freiburg, Germany

**Keywords:** Epigenetics and plasticity, Personalized medicine

## Abstract

In panic disorder (PD), epigenetic mechanisms such as DNA methylation of candidate genes have been suggested to play a key role at the intersection of genetic and environmental factors. On an epigenome-wide level, however, only two studies in PD patients have been published so far, while to date no study has intra-individually analyzed dynamic epigenetic correlates of treatment-response in PD on a DNA methylome level. Here, an epigenome-wide association study (EWAS) was performed in a sample of 57 PD patients and matched healthy controls using the Illumina MethylationEPIC BeadChip, along with a longitudinal approach assessing changes on the DNA methylome level corresponding to clinical effects of a manualized six-week cognitive-behavioral therapy (CBT) in PD. While no epigenome-wide significant hits could be discerned, top suggestive evidence was observed for decreased methylation in PD at cg19917903 in the Cilia and Flagella Associated Protein 46 (*CFAP46*) gene, and for an increase in methylation after CBT at cg06943668 in the Interleukin 1 Receptor Type 1 (*IL1R1*) gene in treatment responders to CBT. Additional exploratory analyses based on biological validity and a combined statistical/biological ranking point to further new potential PD risk genes such as the *CCL4L1* or *GMNN* genes, and suggest dynamic methylation of, e.g., the *ZFP622* and the *SLC43A2* genes along with response to CBT. These EWAS and first longitudinal epigenome-wide pilot data in PD add to the emerging candidate gene-based body of evidence for epigenetic mechanisms to be involved in PD pathogenesis and to possibly constitute dynamic biological correlates of therapeutic interventions.

## Introduction

Panic disorder (PD) is a frequent and highly debilitating mental disorder with a life-time prevalence of 1–3% and women being affected ~2–3 times more often than men^1^. The pathomechanism of PD is complex involving interactions between environmental influences, psychological mechanisms and genetic factors amounting to an estimated heritability of 48%^2^. While cognitive-behavioral therapy (CBT) and pharmacotherapy are very effective for most patients with anxiety disorders, 20–40% of patients with PD suffer from treatment resistance along with a lower socioeconomic status, poorer quality of life and an increased rate of of suicidal attempts^3^.

In anxiety, affective and stress-related disorders, epigenetic mechanisms such as DNA methylation have been suggested to play a key role at the intersection of genetic and environmental factors in disease pathogenesis as well as in treatment response mediation (see refs. ^4,5^). Specifically for PD, there is preliminary evidence for differential DNA methylation of several candidate genes—i.e., the norepinephrine transporter (*SLC6A2*, *NET*)^6^, the monoamine oxidase A (*MAOA*)^7,8^, the glutamate decarboxylase 1 (*GAD1*)^9^, the corticotropin releasing hormone receptor 1 (*CRHR1*)^10^ and the forkhead-box-protein P3 gene (*FOXP3*)^11^—to be involved in disease pathology (for review see refs. ^5,12,13^). On the methylome level, two recent epigenome-wide association studies (EWAS) in PD using the Infinium HumanMethylation450 BeadChip have suggested hypermethylation of a CpG site in the enhancer region of the homo sapiens headcase homolog (Drosophila) (*HECA*) gene in 49 European PD patients and an independent replication sample^14^, and mostly hypomethylation at 40 CpG sites, in 48 Japanese patients with PD as compared to healthy controls^15^. Finally, in a proof-of-principle, candidate gene-based treatment-epigenetic approach, *MAOA* hypomethylation in patients with PD has been observed to be reversible by a successful cognitive-behavioral psychotherapeutic intervention in two independent samples, with responders showing an increase in *MAOA* methylation after treatment, i.e., ‘normalization’ up to methylation levels in healthy controls^8^. To the best of our knowledge, a longitudinal study on epigenetic correlates of treatment-response in PD on the DNA methylome level has not been published so far.

Thus, in the present study we set out (1) to perform an EWAS in a PD case-control design using the Illumina MethylationEPIC BeadChip covering >90% of the original CpGs contained in the HumanMethylation450 BeadChip as utilized in previous PD EWAS^14,15^ plus an additional ~350,000 CpGs, and (2) to analyze changes on the DNA methylome level along with clinical effects of a six-week CBT in PD for the first time in a longitudinal design. These hypothesis-generating approaches are expected to allow for identification of yet unidentified differential DNA methylation in PD, and to potentially reveal epigenetic mechanisms of action of therapeutic interventions based on fear extinction thereby informing clinicians’ treatment decisions towards a more individualized therapy in anxiety disorders.

## Patients and methods

### Samples

Fifty-seven PD patients were recruited at the Department of Psychiatry, Psychosomatics and Psychotherapy, University of Wuerzburg, Germany, within project C02 of the Collaborative Research Center SFB-TRR-58 ‘Fear, Anxiety, Anxiety Disorders’ funded by the German Research Foundation. Caucasian background of all participants was ascertained for at least two preceding generations. PD diagnosis was made on the basis of a structured clinical interview (SCID-I) by experienced psychiatrists and/or clinical psychologists. Apart from bipolar disorder, psychotic disorders, current alcohol dependence, current abuse or dependence on benzodiazepines and other psychoactive substances, comorbid axis I diagnoses were allowed if PD was the primary diagnosis (for details see Table 1). Current or previous severe internal or neurological somatic illnesses, pregnancy, excessive alcohol (>15 glasses of alcohol per week) or nicotine (>20 cigarettes per day) consumption and illegal drugs including cannabis (assessed by urine toxicology) were exclusion criteria.

**Table 1:** Sample characteristics.

Characteristics	PD patient group (*N* = 57)	Control group (*N* = 61)	Statistics
Gender distribution (females vs males)	43 vs 14	47 vs 14	*Χ*^2^ = 0.042, *p* = 0.837
Age in years (mean ± SD)	33.96 ± 9.71	33.25 ± 9.18	*t* = 0.162, *p* = 0.872
Comorbidities (yes vs no):			
Agoraphobia	26 vs 31	n.a.	n.a.
Depression	27 vs 30		
Social anxiety disorder	3 vs 54		
Specific phobias	2 vs 55		
Smoking status (smokers vs non-smokers)	18 vs 39	17 vs 44	*Χ*^2^ = 0.041, *p* = 0.839
Smoked cigarettes per day (mean ± SD)	12.17 ± 6.05	9.56 ± 5.68	*t* = −1.288, *p* = 0.207
Psychiatric medication (yes vs no)^a,b^	31 vs 26	n.a.	n.a.
SSRIs	16 vs 41		
SNRIs	5 vs 52		
NaSSA	6 vs 51		
TCA	7 vs 50		
Pregabaline	2 vs 55		
Antipsychotics	2 vs 55		
Zopiclone	1 vs 56		
Benzodiazepines	1 vs 56		

Sample characteristics are shown for the panic disorder (PD) patient group and the healthy control group

Between-group comparisons were carried out by Chi-square tests for dimensional data or Students *t*-tests for categorical data

*n.a.* not applicable*, SSRIs* selective serotonin re-uptake inhibitors, *SNRIs* selective serotonin and norepinephrine re-uptake inhibitors, *NaSSA* noradrenaline and selective serotonin agonists, *TCA* tricyclic antidepressants

^a^Psychiatric medication at baseline

^b^No patient received any other psychoactive medication, and medication remained unmodified during the course of cognitive-behavioral therapy (see section ‘Treatment’)

A control group of 61 healthy subjects matched to the patient group by age, smoking status and number of smoked cigarettes per day was recruited at the Department of Psychiatry, Psychosomatics and Psychotherapy, University of Wuerzburg, Germany. Current and/or lifetime mental axis I disorder were excluded by experienced psychologists based on the Mini International Neuropsychiatric Interview (MINI) according to DSM-IV criteria. Exclusion criteria corresponded to those applied to the patient sample as listed above. For detailed sample characteristics as well as between-group comparisons please refer to Table 1.

This study was approved by the ethical committee of the University of Wuerzburg, Germany, and conformed to the ethical principles of the Helsinki Declaration. Written informed consent was obtained from all participants.

### Treatment

All 57 PD patients were treated for approximately six weeks in an outpatient clinical setting with six semi-standardized sessions according to a shortened version of the exposure-based CBT manual as applied in the ‘Mechanisms of Action for CBT’ (MAC) study within the network ‘Improving the Treatment of Panic Disorder’ funded by the German Federal Ministry of Education and Research (BMBF)^8,16^. Data from 51 patients having finished treatment were available for intra-individual longitudinal analyses. Psychotherapy was delivered by experienced graduate or clinical psychologists after having accomplished a training workshop on this manual. Therapists were involved in weekly supervision during the course of the study to ensure therapy integrity. The first three sessions lasting ~90 min each were conducted within 2 to 3 weeks and covered psychoeducational information (e.g., physiological, mental and behavioral components of anxiety, vicious circle of anxiety, vulnerability-stress model). The subsequent block of three sessions within the remaining 3–4 weeks comprised interoceptive exercises (e.g., hyperventilation, straw breathing, running). Exposure exercise sessions (100–240 min per session) were conducted approximately once a week and were accompanied by individualized homework. A concluding session focused on therapeutic gains, individual plans for continued exposure exercises and relapse prevention. Medication (*N* = 31 patients; see Table 1) had to be stable for at least two weeks before inclusion into the study and remained unchanged throughout the course of the study. Also, the quantity of smoked cigarettes was kept constant during the course of therapy.

As the primary indicator of disease severity and treatment response, respectively, the clinician-rated Hamilton Anxiety Rating Scale (HAM-A)^17^ was ascertained before (T0) and after (T1) therapy. Data on HAM-A score at T0 and T1 were available for 47 patients. Patients showing at least 50% reduction in HAM-A score (T1-T0) were defined as responders (*N* = 20), all other patients were defined as non-responders (*N* = 27). There was no difference regarding sex, age, smoking behavior, comorbidity with agoraphobia, depression, social anxiety disorder and specific phobias between responders and non-responders (data not shown; all *p* > .05).

### Blood sampling

In the patient sample, EDTA blood was sampled at baseline (T0) and after treatment (T1). In healthy controls, EDTA blood was collected at T0 for the case-control comparison. DNA was isolated using the FlexiGene DNA Kit (QIAGEN, Hilden, Germany) and stored at −80 °C until further processing. In addition, EDTA blood was analyzed for individual differential blood cell count at the Centre of Laboratory Medicine, University of Wuerzburg, Germany.

### DNA methylation analyses

Aliquots of genomic DNA (250 ng) were treated with sodium bisulfite by means of the EZ-96 DNA Methylation Kit (ZymoResearch, Freiburg, Germany). The Infinium MethylationEPIC Kit was used to quantify DNA methylation at ~865,000 sites (Illumina, San Diego, USA). Hybridization and processing were performed according to the manufacturer’s instructions at the Genome Analysis Centre (GAC), Helmholtz Zentrum München GmbH, Munich, Germany.

### Quality control and preprocessing of DNA methylation data

For processing and quality control of the raw methylation data, a customized version of the CPACOR pipeline^18^ was used for quality control, data normalization and calculation of relative DNA methylation values, calculating principal components of the control probes for adjustment and exclusion of outliers based on the Inter-Quartile-Range. The threshold for the sample call rate was set to 0.93. White blood cell type (WBC) sub-populations were estimated based on 100 CpG sites by the Houseman method^19^ as implemented in the minfi R package^20^. Comparison of estimated and measured white blood cell sub-subpopulations in 61 patients and 65 controls showed high concordance, and therefore estimated WBC counts were used for adjustment due to completeness. One sample discordant for reported and estimated sex, based on CpGs on the X- and Y-chromosome, was excluded from analyses. Additionally, quality control based on principal component analyses of the control probes was conducted to detect samples with measurement failures. The complete preprocessing pipeline is available on github (https://github.com/genepi-freiburg/Infinium-preprocessing).

In total, DNA methylation data from 56 PD patients and 60 controls were available for analysis after data pre-processing and quality control, including 56 matched case-control pairs and 47 patients with multiple measures to study changes in DNA methylation and treatment response quantified via the HAM-A.

### Epigenome-wide association analyses

EWAS was carried out using linear regression models and *t*-tests. The first three principal components of the control probes and estimated WBC counts for six cell types (CD8 T cells, CD4 T cells, natural killer cells, B-cells, monocytes, granulocytes) were used in a linear regression as predictors to adjust the relative DNA methylation values for technical biases and white blood cell composition. Using two-sided t-tests the mean of these adjusted DNA methylation values (β-values) was compared at each CpG site between matched cases and controls as well as pre- and post-therapy. Analyses evaluating change in DNA methylation during CBT were stratified according to HAM-A responder status (27 responders, 20 non-responders; see “Treatment” above).

Statistical significance was defined as *p* < 5.77E-8, corresponding to a Bonferroni correction for the 865,859 evaluated CpG sites (0.05/865,859). Associations were reported as suggestive if *p* < 1E-5. Inflation was assessed by the genomic inflation factor lambda^21^ and visual inspection of QQ-plots (see Supplementary Fig. S1). All CpG sites overlapping with single nucleotide polymorphisms (distance to SNP ≤ 5 bp; European ancestry based minor allele frequency ≥ 0.01) were excluded post hoc.

Additionally, given that associated CpG sites do not always have large biological effects, i.e. statistically significantly associated sites might show only very small absolute methylation differences between case and control subjects (Δβ)^22^, CpG sites with highest methylation differences are reported to account for the biological impact of differentially methylated CpG sites. Furthermore, “statistical validity” (*p*-values) and “biological validity” (mean[Δβ]) were used to calculate “combined ranks” according to Dempster et al.^23^. In this effort, all analyzed CpG sites were ranked according to their respective p-value and according to their difference in methylation between groups or time points, respectively. The combined rank was then constructed from the sum of these two ranks. Ties in the combined rank were resolved by priorization of the statistical ranking.

Pathway analyses were conducted for all CpG sites with *p* < 1E-4. GO terms and KEGG pathways were tested for enrichment accounting for the number of CpG sites per gene as implemented in the missMethyl R package^24^. Correction for multiple testing accounted for the 22,633 GO terms and 333 KEGG pathways, respectively, using the method of Benjamini and Hochberg (FDR < 0.05).

## Results

### Case-control analysis

**Fig. 1:**
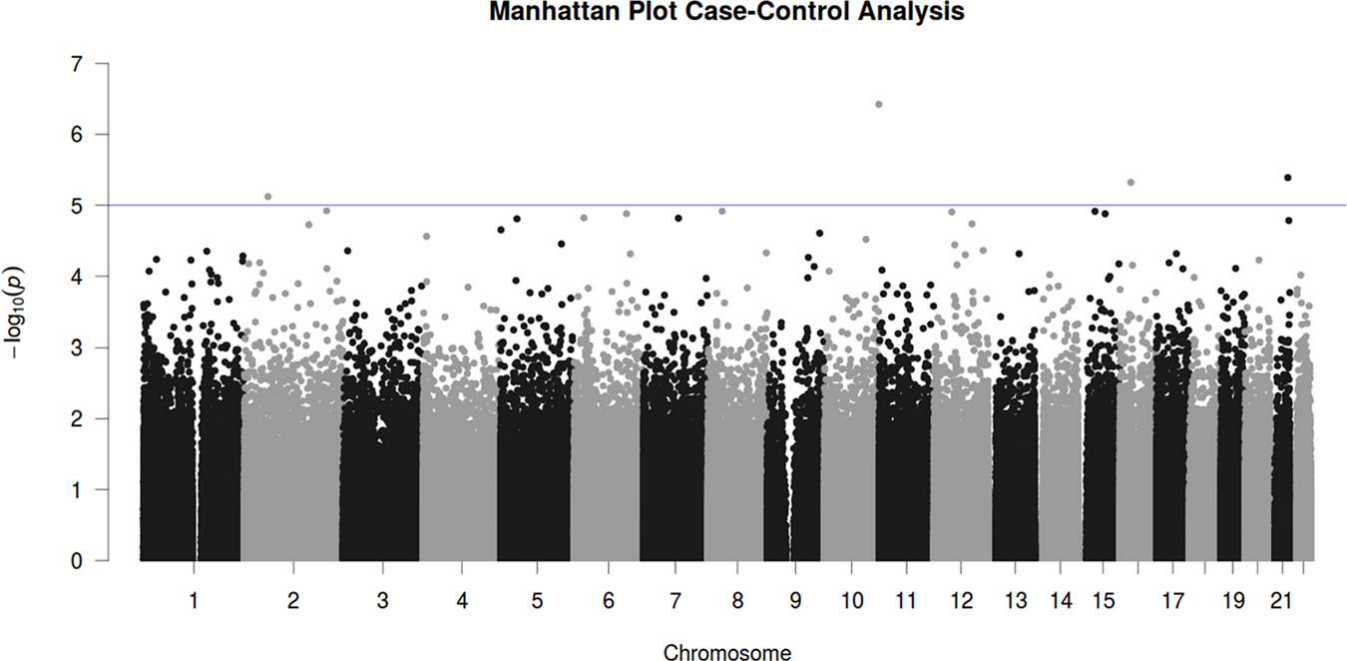
Manhattan plot of the adjusted epigenome-wide panic disorder association study (EWAS) model. The *x* axis shows the chromosomal position, the *y* axis shows *p*-values of the case-control analysis on a -log_10_ scale. The horizontal line indicates the threshold for suggestive sites (*p* = 1E-5).

Epigenome-wide case-control association analyses did not reveal epigenome-wide significant hits (see Fig. 1). However, top suggestive evidence was discerned for decreased methylation in PD patients as compared to healthy controls at cg19917903 in the gene coding for the Cilia and Flagella Associated Protein 46 (*CFAP46*) (see Table 2 for all four hits with *p* < 1E-5).

**Table 2:** Differentially methylated CpG sites with suggestive significance (*p* < 1E-5) in patients with panic disorder as compared to healthy controls.

Rank	CpG site	Nearest gene annotation	Gene name	*p*-value	Mean (Δβ)
1	cg19917903	*CFAP46*	Cilia And Flagella Associated Protein 46	3.8E-07	0.012
2	cg21708468	*RIPK4*	Receptor Interacting Serine/Threonine Kinase 4	4.1E-06	0.011
3	cg06646587	*KDM8*	Lysine Demethylase 8	4.8E-06	−0.012
4	cg14208090	*VRK2*	Vaccinia Related Kinase 2	7.5E-06	0.021

mean (Δβ): Adjusted DNA methylation values (β values) were calculated with a linear model to account for technical differences and blood cell composition. Positive values indicate methylation in patients > methylation in healthy controls, negative values indicate methylation in patients < methylation in healthy controls

When focussing on the “biological validity” (for details, see Methods section), the top-ranked CpG regarding difference in methylation between cases and controls was cg04850148 located within in the intronic region of the C-C motif chemokine ligand 4 like 1 gene (*CCL4L1*) (see Supplementary Table S1 for top ten hits).

When combinedly sorting for statistical as well as biological validity considering both p-values and Δβ values, the top-ranked CpG regarding differential methylation between cases and controls was cg27583138 located in the promoter region of the Geminin gene (*GMNN*) (see Supplementary Table S1 for the top ten hits and Supplementary Fig. S2 for volcano plot).

Pathway analyses revealed no enrichment of GO terms or KEGG pathways in the top 54 CpG sites (*p* < 1E-04).

### Treatment-response analysis

**Fig. 2:**
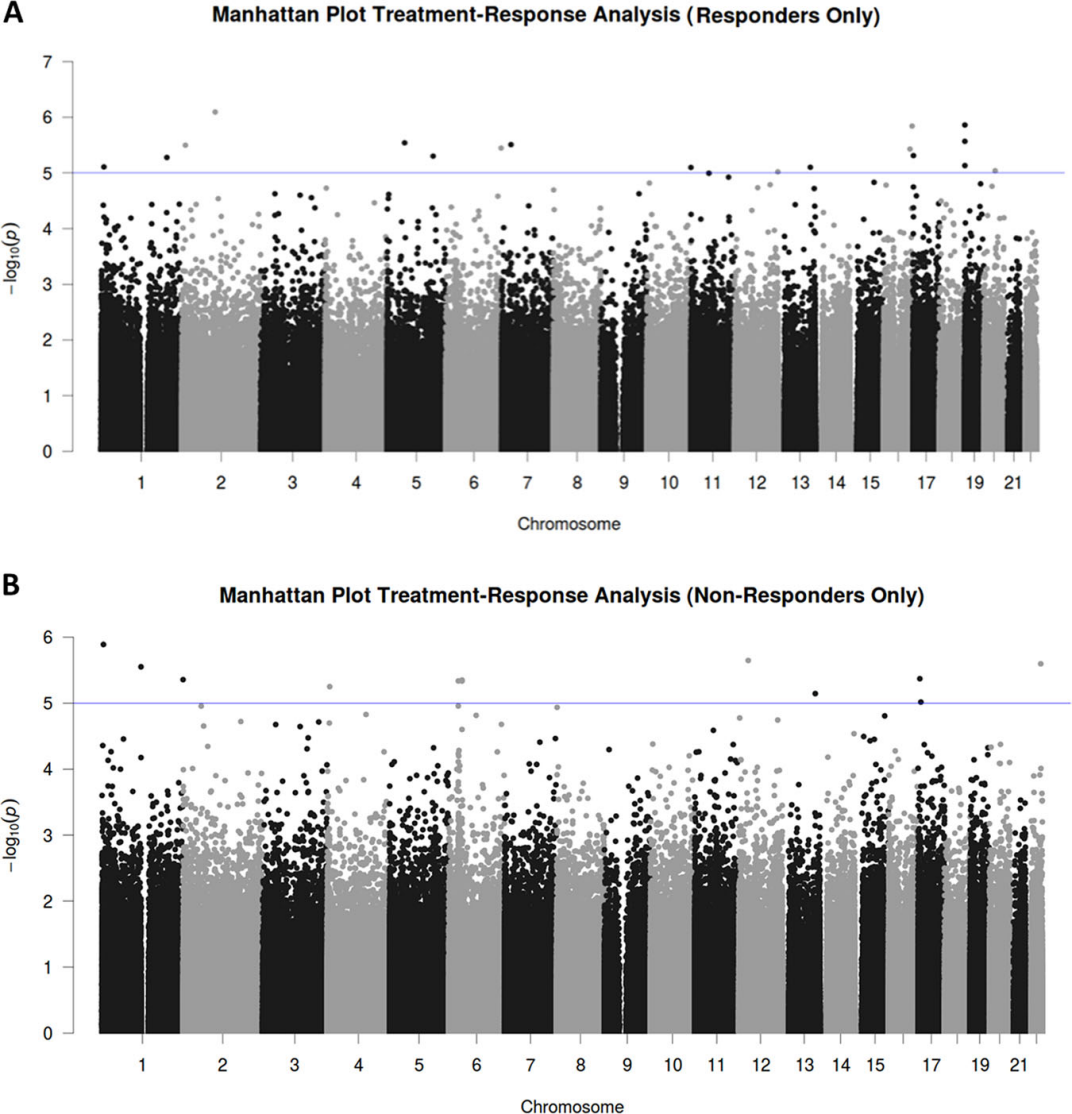
Manhattan plot of the adjusted epigenome-wide treatment-response association study (EWAS) model (stratified by responders and non-responders). The *x* axis shows the chromosomal position, the *y* axis shows *p*-values of the longitudinal analyses on a -log_10_ scale. The horizontal line indicates the threshold for suggestive sites (*p* = 1E-5). **a** Results in the responder stratum and **b** in the non-responder stratum.

When analyzing longitudinal changes in epigenome-wide DNA methylation in the patient sample stratified for responders and non-responders to CBT, top suggestive evidence was observed for an increase in methylation from T0 to T1 at cg06943668 in the gene coding for the Interleukin 1 Receptor Type 1 (*IL1R1*) in treatment responders but not in non-responders (see Fig. 2; see Table 3 for all 18 hits with *p* < 1E-5 in the samples of responders and non-responders, respectively).

**Table 3:** Pre- (T0) to post-therapy (T1) differentially methylated CpG sites with suggestive evidence (*p* < 1E-5) in the patients with panic disorder stratified for responders and non-responders to a six-week cognitive-behavioral therapy.

Rank	CpG site	Nearest gene annotation	Gene name	*p*-value	Mean (Δβ)
*Treatment responders*					
1	cg06943668	*IL1R1*	Interleukin 1 Receptor Type 1	8.0E-07	0.015
2	cg22838050	*MED16*	Mediator Complex Subunit 16	1.4E-06	0.044
3	cg11540895	*JPH3*	Junctophilin 3	1.4E-06	0.015
4	cg09670971	*MED16*	Mediator Complex Subunit 16	2.7E-06	0.051
5	cg22021794	*ESM1*	Endothelial Cell Specific Molecule 1	2.9E-06	−0.019
6	cg13560901	*TRIL*	TLR4 Interactor With Leucine Rich Repeats	3.1E-06	0.016
7	cg23155853	*PDIA6*	Protein Disulfide Isomerase Family A Member 6	3.2E-06	−0.022
8	cg03495053	*THBS2*	Thrombospondin 2	3.6E-06	0.034
9	cg24130365	*PKD1L2*	Polycystin 1 Like 2 (Gene/Pseudogene)	3.7E-06	0.023
10	cg22273830	*SLC43A2*	Solute Carrier Family 43 Member 2	4.9E-06	0.080
11	cg15053890	*FGF1*	Fibroblast Growth Factor 1	5.0E-06	0.030
12	cg14085262	*CHI3L1*	Chitinase 3 Like 1	5.3E-06	−0.023
13	cg15765251	*MED16*	Mediator Complex Subunit 16	7.4E-06	0.038
14	cg09468328	*SPSB1*	SplA/Ryanodine Receptor Domain And SOCS Box Containing 1	7.8E-06	0.010
15	cg27447219	*STK24*	Serine/Threonine Kinase 24	7.9E-06	0.008
16	cg09929763	*B4GALNT4*	Beta-1,4-N-Acetyl-Galactosaminyltransferase 4	8.0E-06	−0.037
17	cg02256614	*DLGAP4*	DLG Associated Protein 4	9.1E-06	−0.012
18	cg25502233	*GALNT9*	Polypeptide N-Acetylgalactosaminyltransferase 9	9.6E-06	−0.020
*Treatment non-responders*					
1	cg20794208	*MIR4417*	MicroRNA 4417	1.3E-06	−0.049
2	cg04403371	*FAR2*	Fatty Acyl-CoA Reductase 2	2.3E-06	−0.032
3	cg01128923	*WNT7B*	Wnt Family Member 7B	2.5E-06	0.020
4	cg20485607	*ZNF697*	Zinc Finger Protein 697	2.8E-06	0.062
5	cg17003694	*NLRP1*	NLR Family Pyrin Domain Containing 1	4.3E-06	0.011
6	cg24582809	*SH3BP5L*	SH3 Binding Domain Protein 5 Like	4.4E-06	0.028
7	cg03644281	*NFYA*	Nuclear Transcription Factor Y Subunit Alpha	4.5E-06	−0.056
8	cg07134666	*ZFP57*	ZFP57 Zinc Finger Protein	4.6E-06	0.057
9	cg04346459	*NFYA*	Nuclear Transcription Factor Y Subunit Alpha	4.6E-06	−0.066
10	cg06068179	*AFAP1*	Actin Filament Associated Protein 1	5.6E-06	0.059
11	cg01641908	*LOC101927437*	Uncharacterized LOC101927437, RNA gene	7.2E-06	−0.012
12	cg15905656	*CCDC42*	Coiled-Coil Domain Containing 42	9.6E-06	0.017

mean (Δβ): Adjusted DNA methylation values (β values) were calculated with a linear model to account for technical differences and blood cell composition. Positive values indicate an increase in methylation from T0 to T1, negative values indicate a decrease in methylation from T0 to T1. Treatment responders and non-responders were defined according to pre- to post-therapy change in Hamilton Anxiety Rating Scale (HAM-A) scores (see “Treatment”)

Additionally, when considering “biological validity”, cg18441082 located in the promoter region of the Zinc Finger Protein 622 (*ZFP622*) gene was the top-ranked CpG with regard to DNA methylation changes from T0 to T1 in treatment responders, but not in non-responders (see Supplementary Table S2 for top ten hits in the samples of responders and non-responders, respectively).

When combinedly sorting for both statistical and biological validity, the first-ranked differentially methylated CpG site from pre- to post-therapy in treatment responders was cg22273830 located in the intronic region of the Solute Carrier Family 43 Member 2 (*SLC43A2*) gene, transcript variants 1 and 2 (see Supplementary Table S2 for top ten hits in the samples of responders and non-responders, respectively, and Supplementary Fig. S3 for volcano plot).

Applying pathway analyses, no enrichment was discerned in the top 116 CpG sites (*p* < 1E-4) of the regression analysis in responders. In non-responders, pathway analyses revealed that the top 90 CpG sites (*p* < 1E-4) were enriched for the KEGG pathway “viral protein interaction with cytokine and cytokine receptor” (4 of 97 genes; *p* = 3E-5).

### In silico functional analyses

An online in silico analysis using BECon^25^ (available at https://redgar598.shinyapps.io/BECon/, as consulted online on 8 April 2019) revealed no or only weak blood-brain methylation correlations for *IL1R1* cg06943668, the hit with the lowest *p*-value with regard to changes from T0 to T1 in CBT responders (BA 10, anterior prefrontal cortex: *r*_*s*_ = 0.06; BA 20, inferior temporal gyrus: *r*_*s*_ = −0.27; BA 7, parietal cortex: *r*_*s*_ = 0.05). The top-ranked CpG derived from case-control comparison *CFAP46* cg19917903 is not contained in the BECon database and thus could not be evaluated. Furthermore, top hits from ranking according to biological validity as well as from combined ranking were evaluated in BECon as follows: *CCL4L1* cg04850148 (BA 10, anterior prefrontal cortex: *r*_*s*_ = 0.30; BA 20, inferior temporal gyrus: *r*_*s*_ = 0.09; BA 7, parietal cortex: *r*_*s*_ = 0.39); *GMNN* cg27583138 (BA 10, anterior prefrontal cortex: *r*_*s*_ = 0.01; BA 20, inferior temporal gyrus: *r*_*s*_ = −0.30; BA 7, parietal cortex: *r*_*s*_ = 0.37), and *ZNF622* cg18441082 (BA 10, anterior prefrontal cortex: *r*_*s*_ = −0.24; BA 20, inferior temporal gyrus: *r*_*s*_ = −0.46; BA 7, parietal cortex: *r*_*s*_ = 0.09). *SLC43A2* cg22273830 is not contained in the BECon database and thus could not be evaluated.

Interrogating the IMAGE-CpG database^26^ (available at https://han-lab.org/methylation/default/imageCpG, as consulted online on 8 April 2019) revealed weak blood-brain correlations for *IL1R1* cg06943668 (*r*_BrainBlood_ = −0.057, *p* = 0.806) and *CFAP46* cg19917903 (*r*_BrainBlood_ = 0.035, *p* = 0.881). However, it has to be noted that for both CpGs methylation levels were comparable in brain and blood with low standard deviations (cg06943668: mean_Brain_ ± SD = 0.933 ± 0.022, mean_Blood_ ± SD = 0.954 ± 0.010; cg19917903: mean_Brain_ ± SD = 0.961 ± 0.013, mean_Blood_ ± SD = 0.951 ± 0.016). The IMAGE-CpG database was furthermore used to evaluate top hits from “biological” and “combined” ranking: *CCL4L1* cg04850148 (*r*_BrainBlood_ = 0.448, *p* = 0.043; mean_Brain_ ± SD = 0.314 ± 0.126, mean_Blood_ ± SD = 0.329 ± 0.120), *GMNN* cg27583138 (*r*_BrainBlood_ = 0.481, *p* = .029; mean_Brain_ ± SD = 0.088 ± 0.059, mean_Blood_ ± SD = 0.529 ± 0.0118), *ZNF622* cg18441082 (*r*_BrainBlood_ = −0.255, *p* = 0.264; mean_Brain_ ± SD = 0.177 ± 0.034, mean_Blood_ ± SD = 0.187 ± 0.023), and *SLC43A2* cg22273830 (*r*_BrainBlood_ = 0.394, *p* = 0.079; mean_Brain_ ± SD = 0.699 ± 0.069, mean_Blood_ ± SD = 0.395 ± 0.209).

## Discussion

The present study applied a hypothesis-generating EWAS approach in an effort to identify (1) yet unknown differential DNA methylation associated with PD in a case-control approach and (2) possibly mechanistically relevant DNA methylation correlates of response to CBT.

### Case-control association study

While no significant epigenome-wide methylation differences could be detected, top suggestive evidence was discerned for differential methylation at CpG site cg19917903 in intron 25 of the Cilia and Flagella Associated Protein 46 gene (*CFAP46*, located on chromosome 10q26.3), with PD patients displaying lower methylation as compared to healthy controls. *CFAP46* is expressed in the brain, particularly the amygdala, the anterior cingulate cortex, the frontal cortex, the basal ganglia, the hippocampus, the hypothalamus, the cerebellum and the substantia nigra^27^ (GTEx portal, available online at www.gtexportal.org; Human Protein Atlas, available online at www.proteinatlas.org, as consulted online on 08^th^ April 2019). *CFAP46* is part of the central apparatus of the microtubule-based cytoskeleton of the cilium. In the brain, motile cilia are found in ependymal cells lining the ventricles and some choroid plexus cells propelling the cerebrospinal fluid, while immotile, i.e. primary cilia exist in neural stem cells, neurons and astrocytes. Neuronal primary cilia serve a variety of purposes from motility to sensing of extracellular mechanical and chemical cues as well as signal production via the generation of extracellular vesicles (for review see ref. ^28^). Cilia are known to be essential for neurogenesis and central nervous system development and related disorders (see refs. ^29–31^). A role in neuronal function related to mental disorders is suggested by studies showing elongation of primary cilia in the mouse brain in response to lithium treatment^32^ and a diminished primary cilia formation in schizophrenia and bipolar disorder^33^. The present findings for the first time provide suggestive support for a potential role of cilia—and particularly of altered *CFAP46* DNA methylation—in the pathogenesis of PD.

Additional exploratory analyses based on biological validity and a combined statistical/biological ranking point to further new potential PD risk genes such as the C-C motif chemokine ligand 4 like 1 gene (*CCL4L1*), a cytokine gene, and the *GMNN* gene, coding for Geminin, a DNA replication inhibitor highly conserved across species and involved in cellular proliferation and neural differentiation^34^.

### Longitudinal psychotherapy-epigenetic study

Analysis of DNA methylation changes on methylome level along with clinical effects of a six-week cognitive-behavioral psychotherapy in PD did not reveal a hit reaching epigenome-wide significance. However, in patients responding to CBT there was top suggestive evidence for methylation at CpG site cg06943668 in intron 1 of the Interleukin 1 Receptor Type 1 (*IL1R1*) gene located on chromosome 2q11.2-q12.1 to increase after treatment, which could not be observed in non-responders. *IL1R1* is expressed in the brain (GTEx portal, available online at www.gtexportal.org; Human Protein Atlas available online at www.proteinatlas.org, as consulted online on 8 April 2019) and encodes a receptor for the pro-inflammatory cytokines interleukin-1 alpha (IL-1 alpha) and beta (IL-1 beta). The present finding of increasing *IL1R1* methylation at cg06943668 along with treatment response—and thus, given the location of cg06943668 in intron 1, potentially decreased *IL1R1* expression (cf refs. ^35,36^)—is in accordance with the existing body of literature suggesting an anxiety-increasing effect of IL-1 and resilience towards anxiety in the absence of *IL1R1*, respectively: In the rat model, intracerebroventricular infusion of IL-1 beta showed rapid diffusion into the amygdala, a core region of anxiogenesis^37^, and gene expression analysis after endotoxin administration revealed an increase in *Il1b* expression in the rat amygdala related to an increase in anxiety-like behavior in the open-field test^38^. In patients with PD and generalized anxiety disorder, significantly elevated plasma IL-1 alpha and beta concentrations were observed as compared to healthy controls^39–41^. Additionally, higher levels of IL-1 beta have predicted non-response to fluoxetine treatment in children with anxiety or depressive disorders^42^. Reciprocally, *Il1r1* knock-out mice spent significantly more time in the open arms of the elevated plus-maze than wild-type animals suggesting a decreased anxiety-like behavior in the absence of Il1r1^43,44^. In a similar vein, anxiety-like behavior as determined using the open-field activity and light/dark preference tests did not develop in *Il1r1* knock-out mice in response to repeated social defeat^45^ or in endothelial-specific *Il1r1* knock-down mice, respectively^46^. Finally, adenosine administration increased anxiety-like behavior along with an increase in IL-1 beta activation in the mouse brain, while *Il1r1* knock-out mice were resistant to adenosine-induced anxiety-like behavior^47^. The present findings suggest a potentially immunomodulatory effect of successful cognitive-behavioral psychotherapy in PD in terms of altered *IL1R1* DNA methylation.

Additional exploratory analyses based on biological validity or a “combined rank” suggest dynamic methylation of several other genes to be potentially involved in treatment response mechanisms of PD, among them the Zinc Finger Protein 622 (*ZFP622*) gene and the *SLC43A2* gene coding for the essential amino acid transporter LAT4, which have not been implicated in mental disorders before.

In CBT non-responders, analysis of DNA methylation changes on methylome level did not discern a hit reaching epigenome-wide significance. However, a pathway analysis revealed overlap of the CpG sites with *p* < 1E-4 with four of 97 genes contained in the KEGG pathway “viral protein interaction with cytokine and cytokine receptor” (*p* < 3E-5). This finding might point towards (neuro)immunological processes—with modulatory effects of particularly cytokines—to be involved in anxiety and possibly also non-response to CBT as previously suggested by Hou and Baldwin^48^, which should be confirmed in future independent replication studies.

In sum, these first longitudinal epigenome-wide findings in PD add to the emerging candidate gene-based body of evidence for epigenetic mechanisms possibly constituting dynamic biological correlates of therapeutic interventions focusing on fear extinction (cf refs. ^49,50^) by complementing previous studies reporting *MAOA* methylation levels to significantly increase along with response to CBT in PD^8^ and acrophobia^51^ as well as dynamic serotonin transporter (*5-HTT, SLC6A4*) and FK506 binding protein 5 (*FKBP5*) gene methylation to correlate with remission status after CBT in a sample of children with mixed primary anxiety disorder diagnoses^52,53^.

### Limitations

Despite several strengths such as high clinical and demographic homogeneity of the present patient sample, strict inclusion/exclusion criteria, consideration of several potential confounders of DNA methylation alterations including control for differential WBC counts and psychotropic medication, application of a case-control as well as a longitudinal therapy-epigenetic design and using the EPIC chip for the first time in a PD sample, several limitations have to be considered when interpreting the present results. While within the range of previous EWAS in PD^14,15^, the present sample size is relatively small, particularly for subsamples stratified for gender and comorbidity (e.g., with/without agoraphobia), and potentially underpowered to detect differential DNA methylation on an epigenome-wide significance level. Thus, future studies in large independent samples using a comparable clinical design are warranted to replicate the present findings and to disentangle potentially sexually dimorphic effects or possibly differential epigenetic mechanisms of “pure” PD and agoraphobia. Given that no randomized waiting-list-controlled study design was applied, it cannot be conclusively argued that the presently observed longitudinal changes in methylation are due to psychotherapy effects only. Also, we were not able to replicate previous EWAS results emerging from a European^14^ and a Japanese^15^ sample of PD patients, which might be due to a lack of power in the present, but also in those previous pilot studies. To inform future studies on necessary sample sizes of EWAS in PD patients, we performed power analyses based on the presently observed distributions. With the same design of matched cases and controls (pre-/post-treatment samples) and an intended power of 90%, an independent discovery EWAS based on the EPIC chip would require sample sizes of 77 and 24 pairs (case-control and responder analyses, respectively) to detect the strongest association signals of the present study at a Bonferroni-adjusted significance level. Evaluating all suggestive sites in a replication analysis, the median CpG site in terms of Cohen’s *d* would require an independent cohort of 21 pairs each for case-control and responder analyses, respectively, to have 90% power (alpha = 0.05/#suggestive sites, one sided). Furthermore, differences in sample composition regarding comorbidities with e.g. agoraphobia or depression, somatic illness, smoking behavior or medication status could explain discrepancies across EWAS in PD. Along these lines, despite careful selection of participants and controlling for potential confounders by means of phenotype matching and adjustment for WBC estimates in regression analyses, latent confounders such as diet, physical activity or life events might have influenced the results. Finally, in human studies investigation of DNA methylation is necessarily restricted to peripheral biomaterial since relevant brain tissue cannot be obtained in vivo. Several lines of evidence emerging from both animal and human studies, however, have suggested DNA isolated from peripheral sources to partly function as a viable surrogate biomarker for central processes with a considerable correlation between methylation patterns in blood and brain tissue in rodents and rhesus monkeys as well as brain metabolism of the respective molecule as measured by positron emission tomography in humans, respectively (e.g., refs. ^54–56^.). Also in other disorders, EWAS revealed methylation sites that translate from whole blood to target tissue (e.g., in obesity^57^ or kidney disease^58^). However, differential DNA methylation identified in peripheral biomaterial such as blood does not allow for direct conclusions regarding methylation correlates in brain tissue, especially given that online in silico analyses using the BECon^25^ or IMAGE-CpG^26^ database revealed no or only weak blood-brain methylation correlations for the presently top-ranked hits, which, however, does not necessarily extend to brain regions relevant for anxiety and fear such as the amygdala, the BNST, the anterior cingulate cortex or the insula.

## Conclusion

In summary, the present EWAS—despite not revealing significant hits—suggests differential *CFAP46* methylation to possibly be involved in PD pathogenesis, and *IL1R1* methylation changes to constitute a potential mechanistic correlate of response to CBT in PD. Provided validation in larger independent samples and functional characterization of their biological relevance these findings might foster further investigation into these epigenetic signatures as potential markers of disease risk or—given the present finding of *IL1R1* methylation changes in treatment responders and potential modulatory epigenetic effects in cytokine-related pathways in non-responders—immunomodulatory pharmacological options in treatment-resistant PD for lasting treatment effects (cf ref. ^59^).

## Supplementary information

Supplementary tables

Legend to supplementary figures

Figure S1

Figure S2

Figure S3

## References

[1] Wittchen HU (2011). The size and burden of mental disorders and other disorders of the brain in Europe 2010. Eur. Neuropsychopharmacol..

[2] Hettema JM, Neale MC, Kendler KS (2001). A review and meta-analysis of the genetic epidemiology of anxiety disorders. Am. J. Psychiatry.

[3] Bystritsky A (2006). Treatment-resistant anxiety disorders. Mol. Psychiatry.

[4] Klengel T, Binder EB (2015). Epigenetics of stress-related psychiatric disorders and gene x environment interactions. Neuron.

[5] Schiele MA, Domschke K (2018). Epigenetics at the crossroads between genes, environment and resilience in anxiety disorders. Genes Brain Behav..

[6] Esler M (2006). The neuronal noradrenaline transporter, anxiety and cardiovascular disease. J. Psychopharmacol..

[7] Domschke K (2012). Monoamine oxidase A gene DNA hypomethylation - a risk factor for panic disorder?. Int J. Neuropsychopharmacol..

[8] Ziegler C (2016). MAOA gene hypomethylation in panic disorder-reversibility of an epigenetic risk pattern by psychotherapy. Transl. Psychiatry.

[9] Domschke K (2013). Epigenetic signature of panic disorder: a role of glutamate decarboxylase 1 (GAD1) DNA hypomethylation?. Prog. Neuropsychopharmacol. Biol. Psychiatry.

[10] Schartner C (2017). CRHR1 promoter hypomethylation: An epigenetic readout of panic disorder?. Eur. Neuropsychopharmacol..

[11] Prelog M (2016). Hypermethylation of FOXP3 promoter and premature aging of the immune system in female patients with panic disorder?. PLoS ONE.

[12] Gottschalk MG, Domschke K (2016). Novel developments in genetic and epigenetic mechanisms of anxiety. Curr. Opin. Psychiatry.

[13] Ziegler C, Domschke K (2018). Epigenetic signature of MAOA and MAOB genes in mental disorders. J. Neural Transm..

[14] Iurato S (2017). DNA Methylation signatures in panic disorder. Transl. Psychiatry.

[15] Shimada-Sugimoto M (2017). Epigenome-wide association study of DNA methylation in panic disorder. Clin. Epigenet..

[16] Gloster AT (2011). Psychological treatment for panic disorder with agoraphobia: a randomized controlled trial to examine the role of therapist-guided exposure in situ in CBT. J. Consult Clin. Psychol..

[17] Hamilton M (1959). The assessment of anxiety states by rating. Br. J. Med. Psychol..

[18] Lehne B (2016). Erratum to: A coherent approach for analysis of the Illumina HumanMethylation450 BeadChip improves data quality and performance in epigenome-wide association studies. Genome Biol.

[19] Houseman EA (2012). DNA methylation arrays as surrogate measures of cell mixture distribution. BMC Bioinforma..

[20] Aryee MJ (2014). Minfi: a flexible and comprehensive Bioconductor package for the analysis of Infinium DNA methylation microarrays. Bioinformatics.

[21] Devlin B, Roeder K (1999). Genomic control for association studies. Biometrics.

[22] Assenov Y (2014). Comprehensive analysis of DNA methylation data with RnBeads. Nat. Methods.

[23] Dempster EL (2011). Disease-associated epigenetic changes in monozygotic twins discordant for schizophrenia and bipolar disorder. Hum. Mol. Genet.

[24] Phipson B, Maksimovic J, Oshlack A (2016). missMethyl: an R package for analyzing data from Illumina’s HumanMethylation450 platform. Bioinformatics.

[25] Edgar RD, Jones MJ, Meaney MJ, Turecki G, Kobor MS (2017). BECon: a tool for interpreting DNA methylation findings from blood in the context of brain. Transl. Psychiatry.

[26] Braun PR (2019). Genome-wide DNA methylation comparison between live human brain and peripheral tissues within individuals. Transl. Psychiatry.

[27] Fagerberg L (2014). Analysis of the human tissue-specific expression by genome-wide integration of transcriptomics and antibody-based proteomics. Mol. Cell Proteom..

[28] Lepanto P, Badano JL, Zolessi FR (2016). Neuron’s little helper: The role of primary cilia in neurogenesis. Neurogenesis.

[29] Lee JE, Gleeson JG (2011). Cilia in the nervous system: linking cilia function and neurodevelopmental disorders. Curr. Opin. Neurol..

[30] Lee JH, Gleeson JG (2010). The role of primary cilia in neuronal function. Neurobiol. Dis..

[31] Youn YH, Han YG (2018). Primary cilia in brain development and diseases. Am. J. Pathol..

[32] Miyoshi K, Kasahara K, Miyazaki I, Asanuma M (2009). Lithium treatment elongates primary cilia in the mouse brain and in cultured cells. Biochem. Biophys. Res. Commun..

[33] Munoz-Estrada J, Lora-Castellanos A, Meza I, Alarcon Elizalde S, Benitez-King G (2018). Primary cilia formation is diminished in schizophrenia and bipolar disorder: a possible marker for these psychiatric diseases. Schizophr. Res.

[34] Taouki I (2017). Geminin participates in differentiation decisions of adult neural stem cells transplanted in the hemiparkinsonian mouse brain. Stem Cells Dev..

[35] Suzuki MM, Bird A (2008). DNA methylation landscapes: provocative insights from epigenomics. Nat. Rev. Genet.

[36] Brenet F (2011). DNA methylation of the first exon is tightly linked to transcriptional silencing. PLoS ONE.

[37] Konsman JP, Blond D, Vigues S (2000). Neurobiology of interleukin-1 receptors: getting the message. Eur. Cytokine Netw..

[38] Engler H (2011). Acute amygdaloid response to systemic inflammation. Brain Behav. Immun..

[39] Brambilla F (1994). Plasma interleukin-1 beta concentrations in panic disorder. Psychiatry Res.

[40] Hoge EA (2009). Broad spectrum of cytokine abnormalities in panic disorder and posttraumatic stress disorder. Depress. Anxiety.

[41] Tang Z (2018). Peripheral proinflammatory cytokines in Chinese patients with generalised anxiety disorder. J. Affect Disord..

[42] Amitai M (2016). The relationship between plasma cytokine levels and response to selective serotonin reuptake inhibitor treatment in children and adolescents with depression and/or anxiety disorders. J. Child Adolesc. Psychopharmacol..

[43] Murray CL, Obiang P, Bannerman D, Cunningham C (2013). Endogenous IL-1 in cognitive function and anxiety: a study in IL-1RI-/- mice. PLoS One.

[44] Koo JW, Duman RS (2009). Interleukin-1 receptor null mutant mice show decreased anxiety-like behavior and enhanced fear memory. Neurosci. Lett..

[45] Wohleb ES (2011). beta-Adrenergic receptor antagonism prevents anxiety-like behavior and microglial reactivity induced by repeated social defeat. J. Neurosci..

[46] Wohleb ES (2014). Knockdown of interleukin-1 receptor type-1 on endothelial cells attenuated stress-induced neuroinflammation and prevented anxiety-like behavior. J. Neurosci..

[47] Chiu GS (2014). Adenosine through the A2A adenosine receptor increases IL-1beta in the brain contributing to anxiety. Brain Behav. Immun..

[48] Hou R, Baldwin DS (2012). A neuroimmunological perspective on anxiety disorders. Hum. Psychopharmacol..

[49] Stafford JM, Lattal KM (2011). Is an epigenetic switch the key to persistent extinction?. Neurobiol. Learn Mem..

[50] Whittle N, Singewald N (2014). HDAC inhibitors as cognitive enhancers in fear, anxiety and trauma therapy: where do we stand?. Biochem Soc. Trans..

[51] Schiele MA (2018). Plasticity of Functional MAOA Gene Methylation in Acrophobia. Int J. Neuropsychopharmacol..

[52] Roberts S (2014). Serotonin transporter [corrected] methylation and response to cognitive behaviour therapy in children with anxiety disorders. Transl. Psychiatry.

[53] Roberts S (2015). Hpa axis related genes and response to psychological therapies: genetics and epigenetics. Depress Anxiety.

[54] Provençal N (2012). The signature of maternal rearing in the methylome in rhesus macaque prefrontal cortex and T cells. J. Neurosci..

[55] Wang D (2012). Peripheral SLC6A4 DNA methylation is associated with in vivo measures of human brain serotonin synthesis and childhood physical aggression. PLoS ONE.

[56] Ursini G (2011). Stress-related methylation of the catechol-O-methyltransferase Val158 allele predicts human prefrontal cognition and activity. J. Neurosci..

[57] Wahl S (2017). Epigenome-wide association study of body mass index, and the adverse outcomes of adiposity. Nature.

[58] Chu AY (2017). Epigenome-wide association studies identify DNA methylation associated with kidney function. Nat. Commun..

[59] Michopoulos V, Powers A, Gillespie CF, Ressler KJ, Jovanovic T (2017). Inflammation in fear- and anxiety-based disorders: PTSD, GAD, and beyond. Neuropsychopharmacology.

