# Supplementary material for: The DNA methylome in panic disorder: a case-control and longitudinal psychotherapy-epigenetic study
Source: Transl Psychiatry. 2019 Nov 21;9:314. doi: 10.1038/s41398-019-0648-6 (PMC6872551; doi:10.1038/s41398-019-0648-6)
Supplement: Supplementary file 1 — Supplementary tables [file 41398_2019_648_MOESM1_ESM.docx]

**SUPPLEMENTARY TABLE S1:** Top ten biologically differentially methylated CpG sites (average absolute methylation change, mean [Δβ]) and combined statistical/biological ranks in patients with panic disorder as compared to healthy controls

| **Biological rank** | **CpG site** | **Nearest gene annotation** | **Gene name** | **p-value** | **mean (**Δβ**)** |
| --- | --- | --- | --- | --- | --- |
| 1 | cg04850148 | *CCL4L1* | C-C Motif Chemokine Ligand 4 Like 1 | 0.0170 | 0.1275 |
| 2 | cg18105134 | *PROZ* | Protein Z, Vitamin K Dependent Plasma Glycoprotein | 0.0011 | -0.1262 |
| 3 | cg04798314 | *SMYD3* | SET And MYND Domain Containing 3 | 0.0378 | 0.1194 |
| 4 | cg11012412 | *LOC101929154* | Uncharacterized LOC101929154 | 0.0057 | -0.1112 |
| 5 | cg22852245 | *MCM9* | Minichromosome Maintenance 9 Homologous Recombination Repair Factor | 0.0279 | -0.1096 |
| 6 | cg24851651 | *CCS* | Copper Chaperone For Superoxide Dismutase | 0.0541 | -0.1060 |
| 7 | cg27266060 | *PHYHIP* | Phytanoyl-CoA 2-Hydroxylase Interacting Protein | 0.0005 | 0.1049 |
| 8 | cg16293892 | *LOC101929154* | Uncharacterized LOC101929154 | 0.0084 | -0.0997 |
| 9 | cg26065642 | *FAM66E* | Family With Sequence Similarity 66 Member E | 0.0201 | 0.0980 |
| 10 | cg12012426 | *UVSSA* | UV Stimulated Scaffold Protein A | 0.0987 | 0.0971 |
| **Combined rank** | **CpG site** | **Nearest gene annotation** | **Gene name** | **p-value** | **mean (**Δβ**)** |
| 1 | cg27583138 | *GMNN* | Geminin, DNA Replication Inhibitor | 1.5E-05 | -0.0540 |
| 2 | cg17030231 | *NXN* | Nucleoredoxin | 0.0004 | 0.0938 |
| 3 | cg03689146 | *POM121L12* | POM121 Transmembrane Nucleoporin Like 12 | 0.0002 | -0.0490 |
| 4 | cg27266060 | *PHYHIP* | Phytanoyl-CoA 2-Hydroxylase Interacting Protein | 0.0005 | 0.1049 |
| 5 | cg15352251 | *LINC00992* | Long Intergenic Non-Protein Coding RNA 992 | 0.0001 | -0.0443 |
| 6 | cg21508673 | *PROZ* | Protein Z, Vitamin K Dependent Plasma Glycoprotein | 0.0006 | -0.0463 |
| 7 | cg06897442 | *PCDHB19P* | Protocadherin Beta 19 Pseudogene | 0.0006 | -0.0434 |
| 8 | cg07700233 | *WDR19* | WD Repeat Domain 19 | 0.0010 | -0.0850 |
| 9 | cg18105134 | *PROZ* | Protein Z, Vitamin K Dependent Plasma Glycoprotein | 0.0011 | -0.1262 |
| 10 | cg26624790 | *GNA11* | G Protein Subunit Alpha 11 | 0.0008 | -0.0454 |

Legend to Supplementary Table S1: mean (Δβ): Adjusted DNA methylation values (β values) were calculated with a linear model to account for technical differences and blood cell composition. Positive values indicate methylation in patients > methylation in healthy controls, negative values indicate methylation in patients < methylation in healthy controls.

**SUPPLEMENTARY TABLE S2:** Top ten pre- to post-therapy biologically differentially methylated CpG sites (average absolute methylation change, mean [Δβ]) and combined statistical/biological ranks in patients with panic disorder stratified for responders and non- responders to a six-week cognitive-behavioral therapy

| **TREATMENT RESPONDERS** | | | | | |
| --- | --- | --- | --- | --- | --- |
| **Biological rank** | **CpG site** | **Nearest gene annotation** | **Gene name** | **p-value** | **mean (**Δβ**)** |
| 1 | cg18441082 | ZNF622 | Zinc Finger Protein 622 | 0.2156 | 0.1256 |
| 2 | cg14351440 | MCF2 | MCF.2 Cell Line Derived Transforming Sequence | 0.1569 | -0.1200 |
| 3 | cg10123377 | LYZL4 | Lysozyme Like 4 | 0.0026 | 0.1177 |
| 4 | cg16955800 | NUP35 | Nucleoporin 35 | 0.2809 | -0.1171 |
| 5 | cg27098574 | BCAR1 | BCAR1, Cas Family Scaffold Protein | 0.3750 | 0.1099 |
| 6 | cg18391209 | CAPN8 | Calpain 8 | 0.0420 | 0.0968 |
| 7 | cg26423139 | SLC43A2 | Solute Carrier Family 43 Member 2 | 6.1E-05 | 0.0854 |
| 8 | cg23595710 | TYW1B | TRNA-YW Synthesizing Protein 1 Homolog B | 0.0007 | -0.0802 |
| 9 | cg22273830 | SLC43A2 | Solute Carrier Family 43 Member 2 | 4.9E-06 | 0.0797 |
| 10 | cg11102724 | GM2A | GM2 Ganglioside Activator | 0.0002 | 0.0742 |
| **Combined rank** | **CpG site** | **Nearest gene annotation** | **Gene name** | **p-value** | **mean (**Δβ**)** |
| 1 | cg22273830 | SLC43A2 | Solute Carrier Family 43 Member 2 | 4.9E-06 | 0.0797 |
| 2 | cg09015861 | OR5AS1 | Olfactory Receptor Family 5 Subfamily AS Member 1 | 1.0E-05 | -0.0660 |
| 3 | cg22297055 | SLC43A2 | Solute Carrier Family 43 Member 2 | 1.8E-05 | 0.0666 |
| 4 | cg11076954 | SLC43A2 | Solute Carrier Family 43 Member 2 | 4.2E-05 | 0.0676 |
| 5 | cg09670971 | MED16 | Mediator Complex Subunit 16 | 2.7E-06 | 0.0510 |
| 6 | cg26423139 | SLC43A2 | Solute Carrier Family 43 Member 2 | 6.1E-05 | 0.0854 |
| 7 | cg03192273 | GM2A | GM2 Ganglioside Activator | 5.6E-05 | 0.0688 |
| 8 | cg15187939 | BCYRN1 | Brain Cytoplasmic RNA 1 | 1.9E-05 | 0.0533 |
| 9 | cg03672997 | TAF3 | TATA-Box Binding Protein Associated Factor 3 | 1.5E-05 | 0.0485 |
| 10 | cg22838050 | MED16 | Mediator Complex Subunit 16 | 1.4E-06 | 0.0444 |
| **TREATMENT NON-RESPONDERS** | | | | | |
| **Biological rank** | **CpG site** | **Nearest gene annotation** | **Gene name** | **p-value** | **mean (**Δβ**)** |
| 1 | cg00781875 | SMG5 | SMG5, Nonsense Mediated MRNA Decay Factor | 0.4733 | 0.0814 |
| 2 | cg17917970 | DUSP9 | Dual Specificity Phosphatase 9 | 0.0530 | 0.0690 |
| 3 | cg15173629 | MIR1321 | MicroRNA 1321 | 0.0930 | -0.0672 |
| 4 | cg13883027 | EHBP1 | EH Domain Binding Protein 1 | 2.2E-05 | -0.0671 |
| 5 | cg03885028 | SOWAHD | Sosondowah Ankyrin Repeat Domain Family Member D | 0.3881 | -0.0668 |
| 6 | cg03031357 | SMS | Spermine Synthase | 0.0303 | 0.0661 |
| 7 | cg19723528 | PPIL2 | Peptidylprolyl Isomerase Like 2 | 0.0042 | -0.0660 |
| 8 | cg04346459 | NFYA | Nuclear Transcription Factor Y Subunit Alpha | 4.6E-06 | -0.0659 |
| 9 | cg02261899 | DCAF12L1 | DDB1 And CUL4 Associated Factor 12 Like 1 | 0.0184 | 0.0628 |
| 10 | cg18441082 | ZNF622 | Zinc Finger Protein 622 | 0.6038 | 0.0628 |
| **Combined rank** | **CpG site** | **Nearest gene annotation** | **Gene name** | **p-value** | **mean (**Δβ**)** |
| 1 | cg20485607 | ZNF697 | Zinc Finger Protein 697 | 2.8E-06 | 0.0624 |
| 2 | cg04346459 | NFYA | Nuclear Transcription Factor Y Subunit Alpha | 4.6E-06 | -0.0659 |
| 3 | cg03644281 | NFYA | Nuclear Transcription Factor Y Subunit Alpha | 4.5E-06 | -0.0560 |
| 4 | cg07134666 | ZFP57 | ZFP57 Zinc Finger Protein | 4.6E-06 | 0.0570 |
| 5 | cg13883027 | EHBP1 | EH Domain Binding Protein 1 | 2.2E-05 | -0.0671 |
| 6 | cg08041448 | ZFP57 | ZFP57 Zinc Finger Protein | 1.1E-05 | 0.0510 |
| 7 | cg10098386 | LINC00929 | Long Intergenic Non-Protein Coding RNA 929 | 3.2E-05 | 0.0462 |
| 8 | cg15570656 | ZFP57 | ZFP57 Zinc Finger Protein | 5.9E-05 | 0.0465 |
| 9 | cg02157626 | ZFP57 | ZFP57 Zinc Finger Protein | 7.7E-05 | 0.0461 |
| 10 | cg14519952 | RFLNA | Refilin A | 0.0001 | -0.0477 |

Legend to Supplementary Table S2: mean (Δβ): Adjusted DNA methylation values (β values) were calculated with a linear model to account for technical differences and blood cell composition. Positive values indicate an increase in methylation from T0 to T1, negative values indicate a decrease in methylation from T0 to T1. Treatment responders and non-responders were defined according to pre- to post-therapy change in Hamilton Anxiety Rating Scale (HAM-A) scores (see “Treatment”).
