# Supplementary material for: The DNA methylome in panic disorder: a case-control and longitudinal psychotherapy-epigenetic study
Source: Transl Psychiatry. 2019 Nov 21;9:314. doi: 10.1038/s41398-019-0648-6 (PMC6872551; doi:10.1038/s41398-019-0648-6)
Supplement: Supplementary file 2 — Legend to supplementary figures [file 41398_2019_648_MOESM2_ESM.docx]

**SUPPLEMENTARY FIGURES**

**SUPPLEMENTARY FIGURE S1:** Quantile-Quantile plots

Legend to Supplementary Figure S1: The QQ plots show the -log_10_ p-values of the analysis on the y-axis compared to the expected ones under the null‑hypothesis on the x-axis. The red line indicates the identity.

**SUPPLEMENTARY FIGURE S2:** Volcano plot of combined statistical (-log_10_[p-value]) / biological (mean[Δβ]) ranks in patients with panic disorder as compared to healthy controls

**SUPPLEMENTARY FIGURE S3:** Volcano plot of combined statistical (-log_10_[p-value]) / biological (mean[Δβ]) ranks of pre- to post-therapy differentially methylated CpG sites in patients with panic disorder responding to a six-week cognitive-behavioral therapy
