# Supplementary figures and images for: The DNA methylome in panic disorder: a case-control and longitudinal psychotherapy-epigenetic study
Source: Transl Psychiatry. 2019 Nov 21;9:314. doi: 10.1038/s41398-019-0648-6 (PMC6872551; doi:10.1038/s41398-019-0648-6)

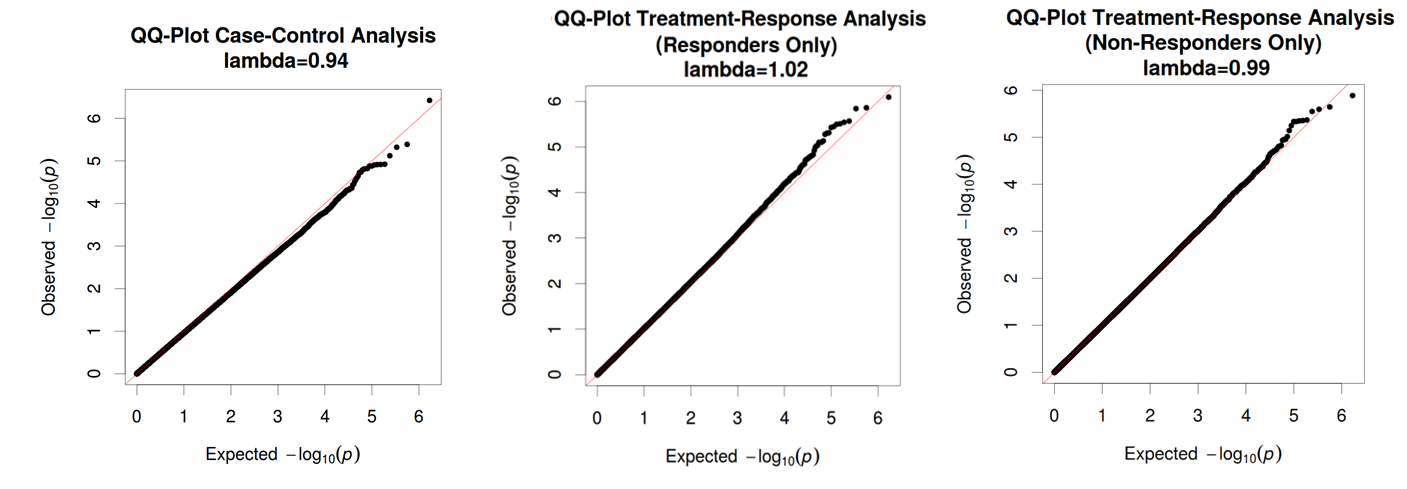

Supplement: Supplementary file 3 — Figure S1 [file 41398_2019_648_MOESM3_ESM.tif]

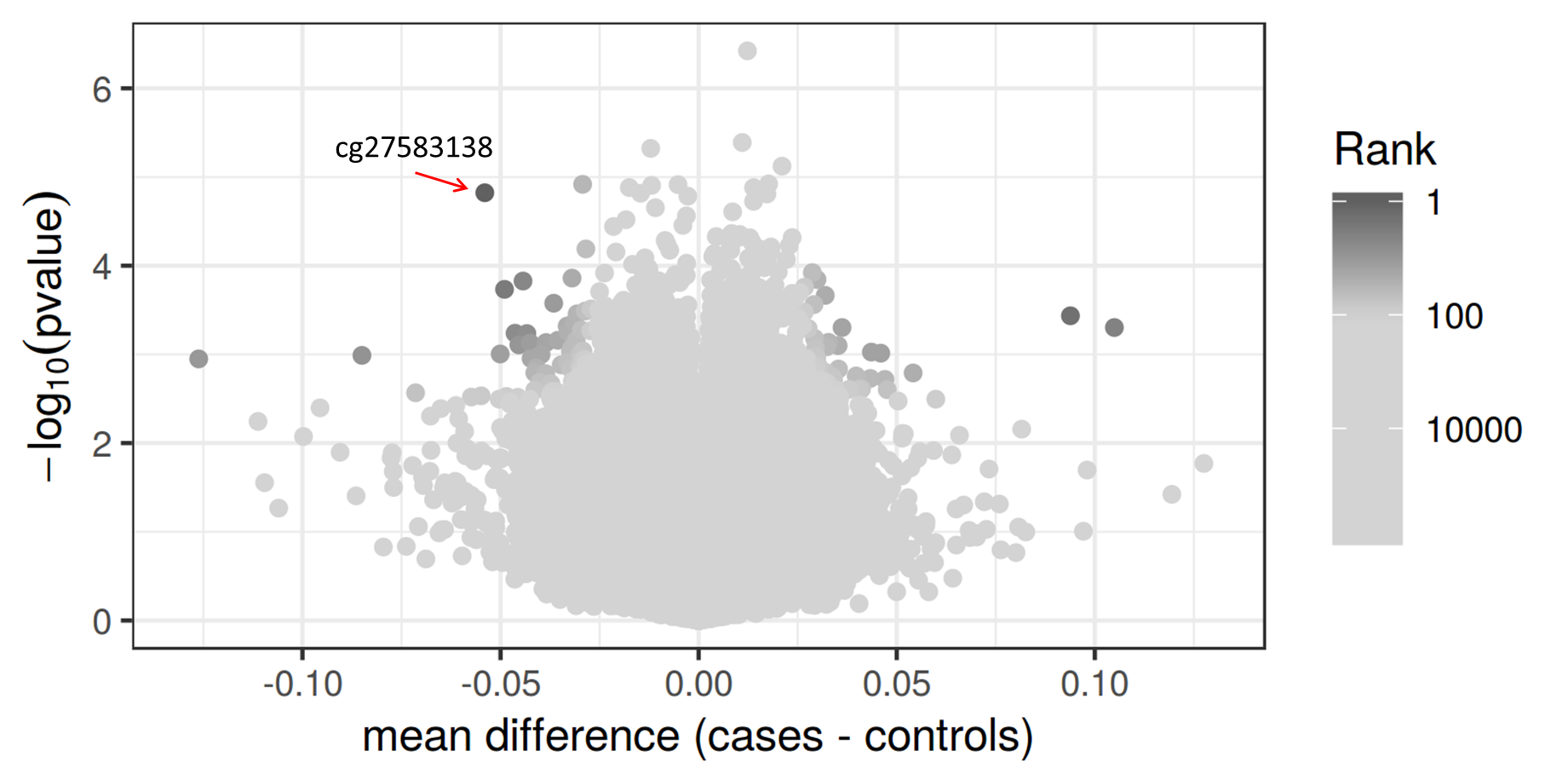

Supplement: Supplementary file 4 — Figure S2 [file 41398_2019_648_MOESM4_ESM.tif]

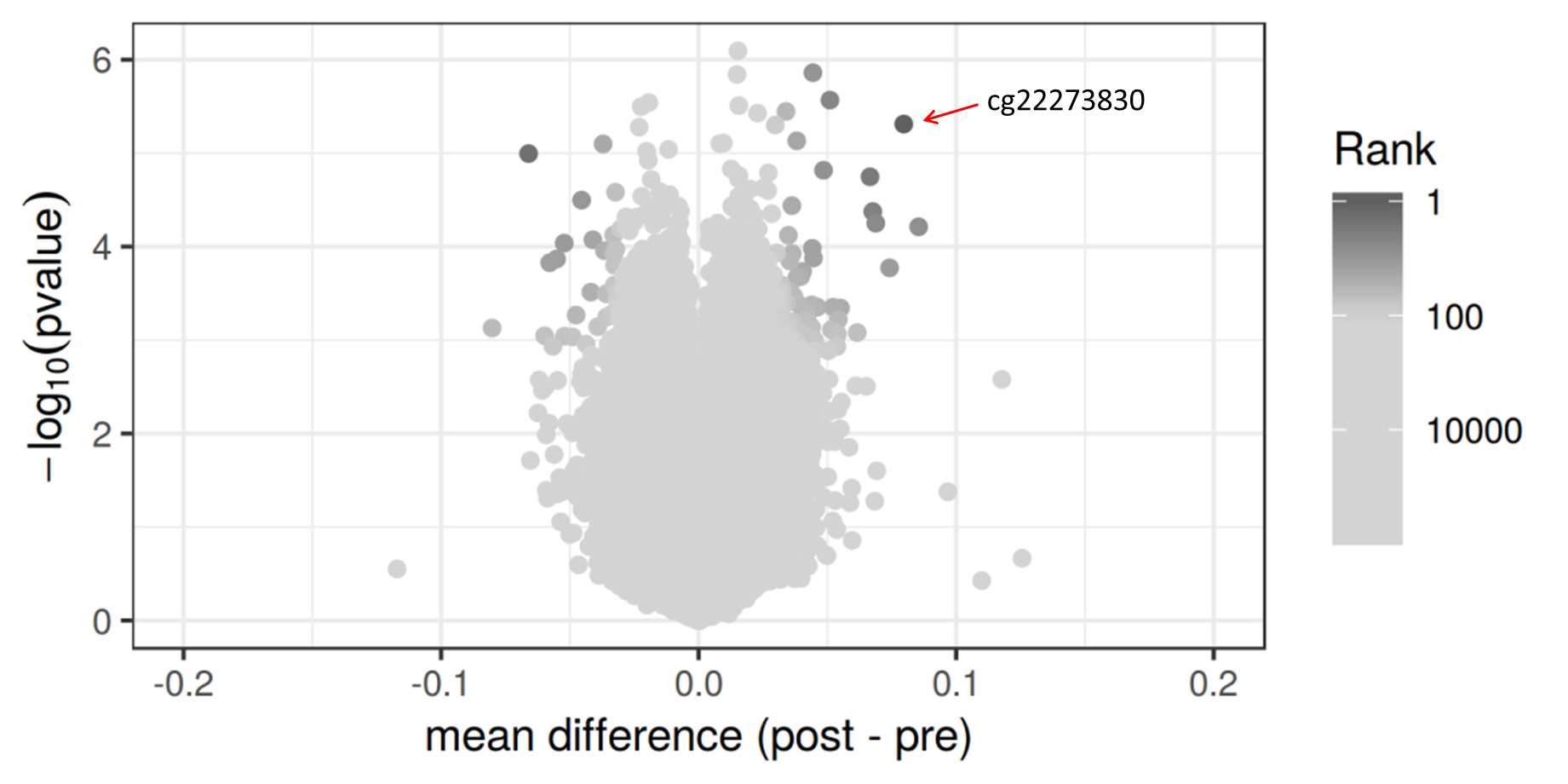

Supplement: Supplementary file 5 — Figure S3 [file 41398_2019_648_MOESM5_ESM.tif]
